# A blockchain-based framework for electronic medical records sharing with fine-grained access control

**DOI:** 10.1371/journal.pone.0239946

**Published:** 2020-10-06

**Authors:** Jin Sun, Lili Ren, Shangping Wang, Xiaomin Yao

**Affiliations:** School of Science, Xi’an University of Technology, Xi’an, Shaanxi, China; Wuhan University, CHINA

## Abstract

In the medical system, the verification, preservation and synchronization of electronic medical records has always been a difficult problem, and the random dissemination of patient records will bring various risks to patient privacy. Therefore, how to achieve secure data sharing on the basis of ensuring users’ personal privacy becomes the key. In recent years, blockchain has been proposed to be a promising solution to achieve data sharing with security and privacy preservation due to its advantages of immutability. So, a distributed electronic medical records searchable scheme was proposed by leveraging blockchain and smart contract technology. Firstly, we perform a hash calculation on the electronic medical data and store the corresponding value on the blockchain to ensure its integrity and authenticity. Then, we encrypt the electronic medical data and store it in the interplanetary file system which is a distributed storage protocol. These operations not only can solve centralized data store of servers of several medical institutions, but also be good at lowering stress from data store and high-frequency access to blockchain. Secondly, the encrypted keyword index information of electronic medical records was stored on the Ethereum blockchain, meanwhile a smart contract deployed in the Ethereum blockchain is used to realize keyword search instead of depending on a centralized third party. Furthermore, we use attribute-based encryption scheme to ensure that only the attributes meeting the access policy can decrypt the encrypted electronic medical records. Finally, our performance analysis and security analysis show that the scheme is secure and efficient.

## 1. Introduction

In recent years, with the development and application of cloud computing technology, the electronic medical records (EMRs) system has shown a trend of doctor-centered development. In this case, one of the most controversial issue is how to ensure the privacy, security, and sharability of EMRs while achieving fine-grained access control. An effective solution is to combine cloud storage, attribute-based encryption (ABE), and searchable encryption technologies. However, this approach has some challenges in terms of privacy, security, and interoperability.

Firstly, in the health care system, the patient’s medical information such as prescriptions, nuclear magnetic resonance image, assay and pathological results are privacy-sensitive and contain all the information related to personal health. Especially when using various encryption mechanisms to protect personal medical records, and the centralized key management adopted may bring a single point of failure, thereby increasing the risk of data exposure. Second, almost all ABE schemes require a trusted entity authority to distribute attribute keys to the system. It is usually assumed that the entity is fully trusted, but it is difficult to find a fully trusted authority in reality. Moreover, for the EMR outsourcing and storing in the third part, some keywords would be fully utilized while other users seek to make a study and analysis for the required data. However, since EMR involves the privacy of users, privacy preserving should be considered during searching process. Furthermore, the identity authentication of the data requester should be taken into account in the keyword searches to prevent identity forgery of illegal users. Finally, cloud storage often plays an important role in data storage environments. To ensure the data is not tampered with, lost, accessed illegally, or stolen arbitrarily, the most common technique is to encrypt shared content before storing it to a cloud server. However, in practice, the cloud server is always regarded as a semi-trusted entity that can perform a small number of search operations and forge some wrong search results back to the user. These erroneous or incomplete personal medical records may mislead users, (such as doctors, research institutes, or other patients) to make incorrect judgments, which could endanger an individual’s life. Therefore, if we create a shared data source for person such as doctors, caregivers, research institutes, and insurance companies to provide the timely, accurate and extensive data and enabling cross-institutional data sharing, it can help health care professionals develop optimal treatment plans. In addition, the secure sharing of EMRs can provide researchers with a broad data set to study disease and to accelerate the development of biomedicine.

Several [1–5] encryption methods have been put forward for these medical data sharing and storage problems, but they are still not enough. Fortunately, a scholar named Satoshi Nakamoto published a new paper [6], “Bitcoin: a peer-to-peer electronic cash system” that expounds the electronic currency theory based on cryptography. After the emergence of Bitcoin, its underlying technology blockchain [7] quickly gained recognition from all walks of life and began to research eagerly. The birth of blockchain technology, with its attractive characteristics such as invariance and decentralization [8, 9] is considered as a powerful fit to provide an appropriate solution to solve these problems and change the centralized storage mode of this medical data. This has attracted widespread attention in academia and industry, including medical [10–12] finance [13] and energy [14–16]. In addition, the blockchain paves the way for building smart contracts [17], which acts as a piece of code on the blockchain and can execute operations once certain criterion be met. More importantly, smart contracts that can be performed autonomously without the need for an honest third party. In addition, the Inter-Planetary File System (IPFS) is a global point-to-point distributed version file system. The combination of IPFS and blockchain allows users to process large amounts of data through IPFS, so that the data itself does not need to be placed on the chain, which not only saves the network bandwidth of the blockchain, but also effectively protects it. As for the security of the data, on the one hand, it can be stored in IPFS after encryption; on the other hand, the distributed sharing of the file can be realized by using the IPFS, which effectively solves the problem that the cloud provider deletes part of the data in order to save storage space in the traditional storage mode. Therefore, the combination of blockchain technology and IPFS can provide a good solution for the above problems.

### 1.1 Our contributions

Based on the above statement, this paper proposed a secure storage and searchable scheme for EMRs with fine-grained access control based on blockchain. Our scheme takes advantage of the blockchain and smart contract technology to implement a decentralized data sharing, on which it gracefully preserve the authenticity and integrity of EMRs. The main contributions of this article are summarized as follows:

1. A point-to-point distributed storage system IPFS was adopted. The medical data files are encrypted and stored in the IPFS to ensure complete EMRs. This eliminates the needs to put the data itself on the chain, which not only saves the network bandwidth of the blockchain, but also make up for the shortcomings of the existing blockchain system in file storage.
2. Using CP-ABE technology and auditing ideas to achieve doctor-centric access control. That is to say, the new scheme allows doctors and patients to set the policy about who has the right to access EMRs for realizing more secure data management and resisting data forgery. Thus, only data requesters who were authorized and their attributes satisfy the access policy in the ciphertext can decrypt the EMRs.
3. Building an encrypted keyword index for shared medical records, in order to implement EMRs’ secure search in a distributed storage system, keyword index information is stored in a blockchain, and smart contract is deployed on the Ethereum blockchain. Once the smart contract is deployed, it will be executed automatically and operated in good faith.
4. An application example of a medical insurance scenario is given. Finally, safety analysis and performance analysis show that our scheme is practical and feasible.

## 2. Related work

Quite a few schemes have been proposed to deal with the vulnerability in current EMRs system. In 2010, Ibraimi et al. [18] applied ABE to the security management of personal health records to achieve flexible access control. Nonetheless, a strict safety proof still has not worked out in this paper. In 2013, Li et al. [19] proposed a novel patient-centric framework and a suite of mechanisms for data access control to personal health records stored in semi-trusted servers. And the paper supported multiple data application scenarios. However, since the cloud server is not completely trusted which sometimes tamper with the received encrypted data, data privacy needs to be further studied.

In 2015, XhafaF et al. [20] proposed a cloud-based medical record system which adopts an ABE scheme to encrypt medical information. These data utilized patient’s condition without leaking definited description of illness and the department in which the doctor is located. In fact, the paper adopts a symmetric encryption scheme, but its implementation must depend on an entirely honest Global Authority to take charge of key management, issuing public parameters for the system and generating secret keys for the doctor, that is one of the main shortcomings of this scheme.

A mobile application for medical data sharing was implemented in work [21], but it was limited to patients and doctors. In addition, the scheme did not support without supporting data sharing with third parties such as insurance companies and medical research centers. Similarly, reference [8] proposed interoperability proof to avoid computational cost. This method uses a single centralized source of trust for transactions to support network consensus. However, the literature fails to achieve fine-grained access control. In 2017, Q. Xia et al. [22] put forward a blockchain-based health data sharing framework that fully solves the access policy challenges related with sensitive information stored in the cloud. The framework is based on a licensed blockchain that only allows the authorized and the invited users to access. In addition, in order to offer a source of medical data, auditing and secure data tracking, the authors used access control and smart contracts mechanisms in their other paper [10] to effectively track data action and to revoke access to violating entities, but none of them achieve the searchability of data. Blockchain is used in [23] to ensure that users have reliable access to electronic health records. Further, the author relies on smart contracts to manage physicians’ electronic medical record usage and focus on theoretical analysis, so the feasibility of the proposed solution has not been confirmed in the actual electronic health records sharing scenario. In 2018, Zhang et al. [11] proposed a data sharing scheme for implementing privacy protection in personal health information (PHI) system through the consortium blockchain. The scheme constructed two kinds of blockchain by designing data structures and consensus mechanisms. On the basis of blockchain, in order to achieve secure search, privacy protection, data security and access control, PHI sharing protocol is proposed by using public key encryption and keyword search technology. However, the scheme does not achieve complete control over the data, such as data revocation, setting data access deadlines, and so on. In 2018, Sun et al. [24] proposed a searchable personal health record framework with fine-grained access control in cloud-fog computing, which adopted CP-ABE to outsource part of the computational operations to fog nodes, effectively reducing the computational burden in cloud storage. However, the implementation of this solution must rely on multiple authorization centers, so the confidentiality of the data cannot be guaranteed. At the same year, Zhang et al. [25] proposed a FHIRChain based on blockchain architecture for sharing clinical data, and demonstrated a distributed application based on FHIRChain, which uses digital health identity to authenticate participants. However, the performance of the FHIRChain prototype is not strict enough, and fine-grained access control is not implemented for the identity management of the group.

In 2019, Kurt et al. [26] offered some ways to modern medical infrastructure by taking advantage of the potential value of the blockchain technology. However, these approaches are only a theoretical explanation and there are no related schemes or specific implementations. In the same year, DINH [27] et al. designed a reliable access control mechanism to manage user access based on a single smart contract to ensure efficient and secure electronic health record sharing, but the cryptographic algorithms involved in data protection were less secure.

## 3. Preliminaries

Next, we give some background knowledge used in this article, such as blockchain, smart contract, IPFS, and some cryptographic tools throughout the whole paper.

### 3.1 Blockchain

In 2008, Satoshi Nakamoto first proposed the concept of blockchain [6], it is called Bitcoin as the underline technology behind the cryptocurrency, which helps to realize peer-to-peer value exchange without centralized third parties. The process about storage, accounting, maintenance, verification and transmission of blockchain data is based on the distributed system structure, using pure mathematical methods rather than central institutions to establish trust relationships among distributed nodes, thus forming a decentralized trusted distributed system. Fundamentally, it is a set of nodes named verifiers or miners that are responsible for maintaining a trusted record for all transactions via a consensus algorithm in a trust-free environment.

**Fig 1:**
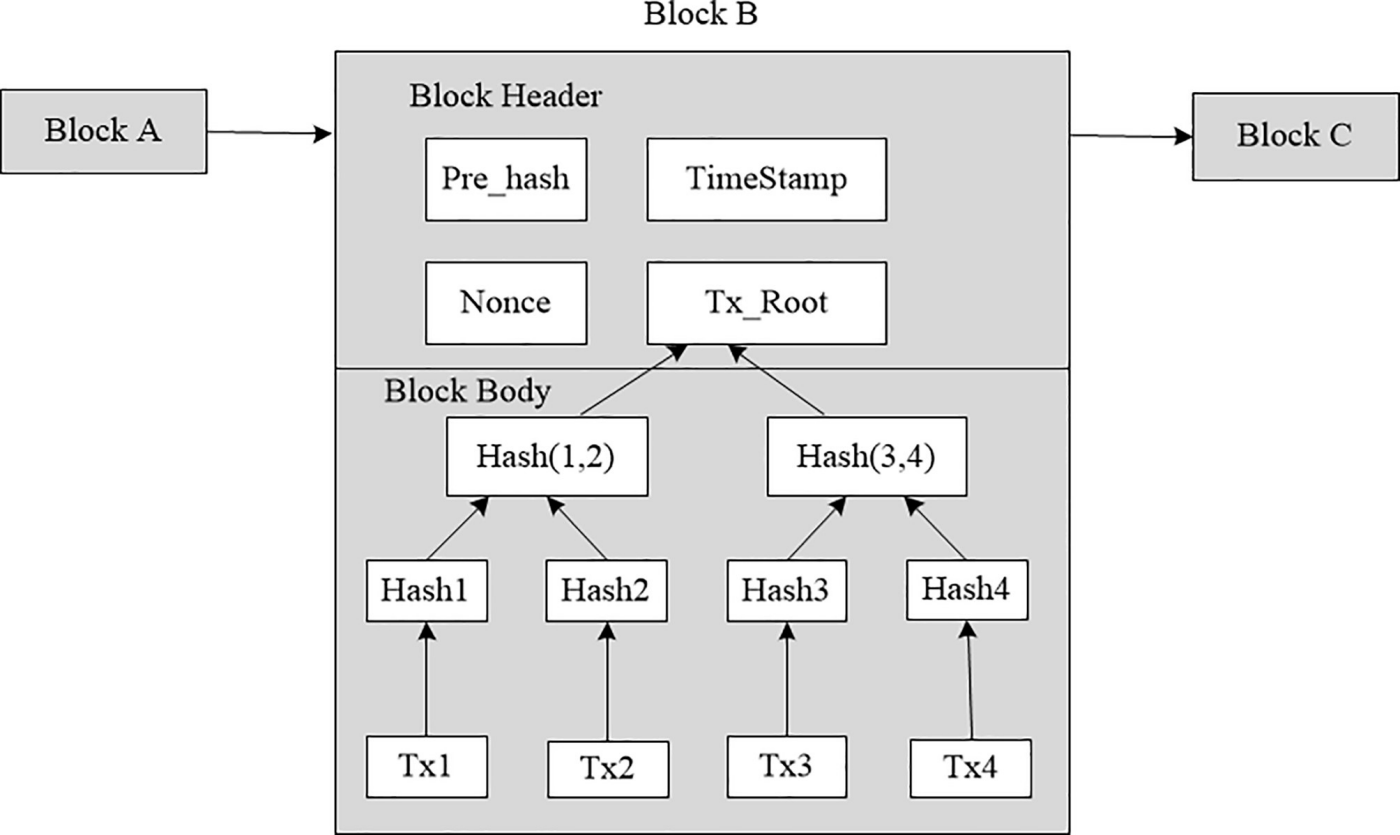
Blockchain structure.

As the name implies, a blockchain is made up of many blocks, among which the block refers to the collection of all information communication data in the system within a period of time. A block is the basic unit that forms blockchain, and each block has a timestamp as its unique mark to ensure the traceability of the blockchain. Each block is divided into two parts: Block Header and Block Body, each block header contains the link pointers of the block headers of the previous block, the Merkle root of the tree-like transaction information, and a timestamp. And the block body records the data information in the network. In this way, the blocks are linked together in chronological order. The blockchain structure is shown in Fig 1.

### 3.2 Smart contract

**Fig 2:**
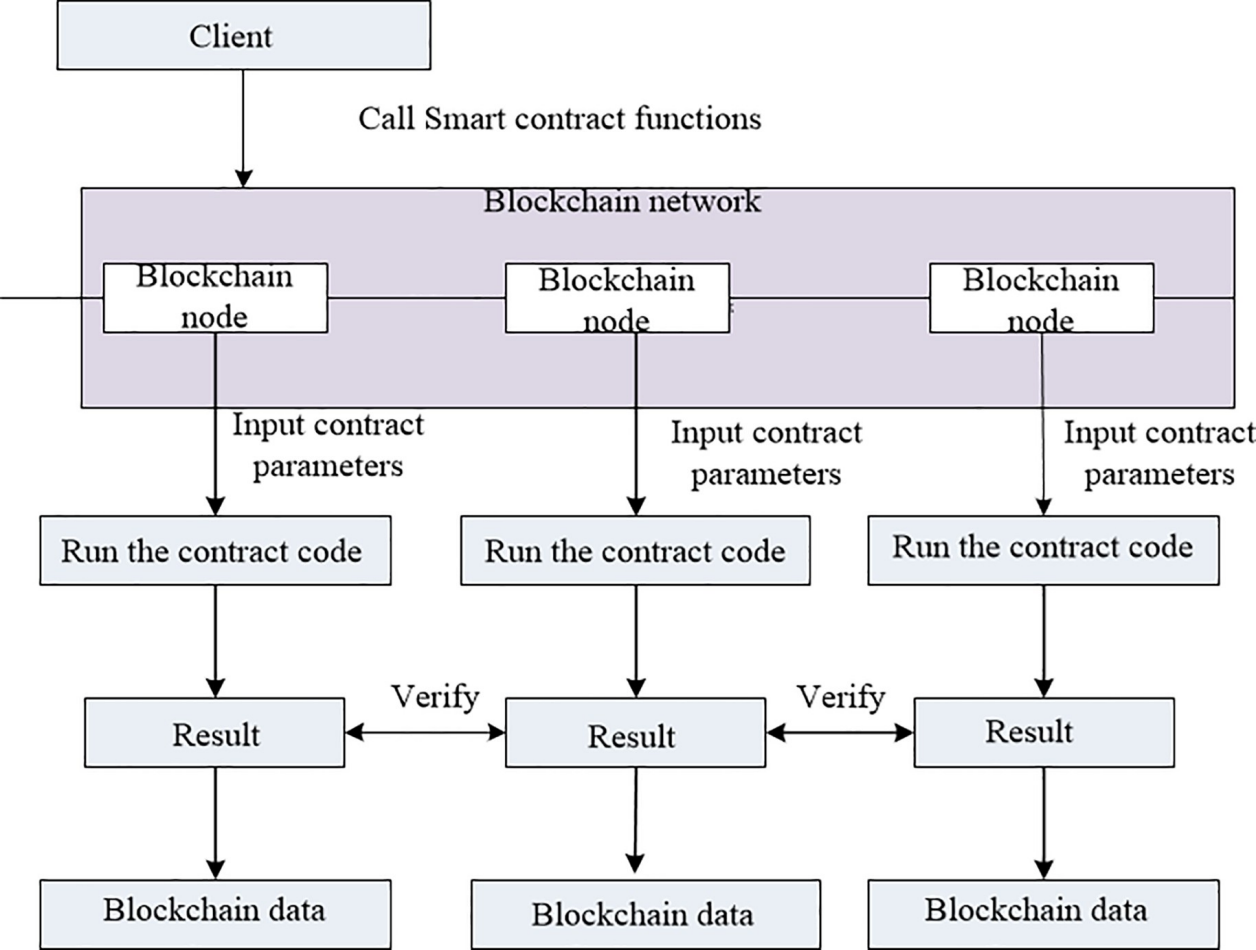
Smart contract operation mode.

A smart contract is a special account with associated data and code. The code is in an Ethereum-specific binary format (i.e., Ethereum Virtual Machine byte code) and deployed by an account to a global database known as blockchain. Smart contracts are an exciting application of blockchain. The smart contract of the blockchain can be understood as a piece of code (contract) that triggers execution when the two parties make a transaction transfer on the blockchain asset. The operation mode is shown in Fig 2. A smart contract usually provides many functions or application binary interfaces (ABIs) that can be used to interact with it. These ABIs can be executed by sending a transaction from an account or a message to another contract. In this paper, the doctor creates a smart contract for storing the keyword index of the patient’s medical records and some related data, in the meantime guaranteeing a fair search. In the absence of a trusted third-party entity, data requester mortgages search fees to smart contracts, and smart contracts help users retrieve data. If the wrong search data is returned, the service fee cannot be charged.

### 3.3 IPFS

IPFS (InterPlanetary File System) [28] is a peer-to-peer data distribution protocol in which nodes came form a distributed file system. When we upload the file to the IPFS network, the system will split the file into N fragments, each fragment generates a hash value, and all the fragment hashes are combined to generate a hash value of the total file. This hash value is the address of the file within the IPFS. When querying a hash to IPFS, IPFS can quickly find the node owning the data and retrieve it by using a distributed hash table, and then use the hash to verify whether it is the correct data. The IPFS network is a fine-grained, distributed and easily federated content distribution network that is common to all data types including images, video streams, distributed databases, operating systems etc, and has fewer storage restrictions.

### 3.4 Cryptographic tools

The main symbols used in the remaining articles are showed in Table 1.

**Table 1:** Symbols table.

Symbol	Descriptions
*U*	Attribute universe
*MSK*	Master key
*PK*	Public key
*uk*	Attributes private key
*m*	The original medical records
*pk*_*i*_,*sk*_*i*_	Public-private key pair of the patient
*pk*_*j*_,*sk*_*j*_	Public-private key pair of the doctor
*AV*_*ij*_	The medical records authentication value
*h*_*location*_	The encrypt medical records’ location
*W*_*m*_	Keywords set
*index*	The index of the keywords
$T\tilde{w}$	Search token

#### 3.4.1 Attribute-based encryption

Attribute-based encryption introduced by Sahai and Waters [29] is considered an expansion of identity-based encryption [30] by treating identity as a set of attributes. So far, there are two types of ABE schemes, namely ciphertext policy attribute-based encryption (CP-ABE) and key policy attribute-based encryption (KP-ABE), which are introduced by Goyal et al [31]. In our scheme, we adopt CP-ABE scheme proposed in to enable the doctor and patient to identify the data requester who have the attribute to decrypt an EMR.

#### 3.4.2 Symmetric searchable encryption [32]

Symmetric searchable encryption involves three entities, data owner, server and data user. The data owner and the data user can be the same person. Assume that the data owner has documents that need to be stored on the private cloud server, he encrypts these documents to generate ciphertext and the corresponding index, and then sends them to the cloud server. When the data user needs to search for a keyword that he interested, he generates a search token of the keyword according to his private key, and send the search token to the cloud server. The cloud server retrieves the corresponding document identity with the received search token and index, and then returns the successfully matched documents to the user. In our scheme, the doctor generates a keyword index of the medical record and stores it in the smart contract. When the data requester needs to search a medical record, he uses his own search private key to generate a search token to call the smart contract. The smart contract first determines the identity of the data requester and then returns the related search result.

#### 3.4.3 Bilinear map [25]

Let $G_{0}$ and $G_{1}$ be two multiplicative cyclic groups of prime order *p* Let *g*_0_ denote one generator of $G_{0}$. A bilinear pairing operation constructed as $e:G_{0}\times G_{0}\to G_{1}$ with the following properties:

1. Bilinearity: For $\forall u,v\in G_{0}$ and all $a,b\in {\mathbb{Z}}_{p}$, *e*(*u*^*a*^,*v*^*b*^) = *e*(*u*,*v*)^*ab*^ holds.
2. Non-degeneracy: *e*(*g*_0_,*g*_0_)≠1.
3. Computability: For all $\forall u,v\in G_{0}$, *e*(*u*,*v*) can be efficiently computed.

#### 3.4.4 General bilinear group [33]

Let *ϕ*_0_, *ϕ*_1_ be two mapping functions ${\mathbb{Z}}_{p}^{+}\to {\left\{0,1\right\}}^{k}$, where ${\mathbb{Z}}_{p}^{+}$ is an additive group, *k*>3 log(*p*). The groups $G_{0}$, $G_{1}$ are defined as $G_{0}=\left\{\phi_{0}\left(\chi \right)|\chi \in {\mathbb{Z}}_{p}\right\}$, $G_{1}=\left\{\phi_{1}\left(\chi \right)|\chi \in {\mathbb{Z}}_{p}\right\}$ respectively, where $G_{0}$ is a general bilinear group, *g* denotes *ϕ*_0_(1), and *g*^*x*^ represents *ϕ*_0_(*x*), *e*(*g*,*g*) denotes *ϕ*_1_(*x*) and *e*(*g*,*g*)^*x*^ represents *ϕ*_1_(*x*).

#### 3.4.5 Access structure

There are several kinds of access structure in ABE scheme, such as AND-gate structure, tree-based structure and threshold structure. In our protocol, we adopt a series of AND gate as our access policy.

Let assume the total number of attributes is *n*, and all attributes be indexed as *U* = {*x*_1_,*x*_2_,⋯,*x*_*n*_}. For every attribute *x*_*i*_∈*U*, (*i* = 1,2,⋯,*n*), *V*_*i*_ = {*v*_*i*,1_,*v*_*i*,2_,⋯,*v*_*i*,*n*_} be a set of possible values of *x*_*i*_, where *n*_*i*_ is the number of the possible values for *x*_*i*_. Then the attribute list *S* for a user is *S* = (*att*_1_,*att*_2_,⋯,*att*_*n*_), where *att*_*i*_∈*V*_*i*_. The access policy in ciphertext is $A=\left(A_{1},A_{2},\cdots ,A_{n}\right)$, where $A_{i}\in v_{i}$. We define the attribute list *S* satisfies the access policy A if and only if $att_{i}=A_{i}$, (*i* = 1,2,⋯,*n*).

#### 3.4.6 Security model

We designed security game to prove the security of our scheme, which depends on the general bilinear group model and the game is semantically secure against an adaptive chosen plaintext attack (IND -CPA).

**IND-CPA game**:
**Initialization**: The adversary A submits an access structure $A^{*}$ that he wishes to challenge it.
**Setup**: The challenger B runs the global setup algorithm to generate the public key *PK* and master key *MSK*, then sends the public key *PK* to adversary A, and holds the master key *MSK* privately.
**Phase1**: A can repeatedly ask the $O_{SK}\left(S\right)$ oracle.
$O_{SK}\left(S\right)$: A can issue queries for private key *SK* by submitting attribute set S.
**Challenge**: A submit two equal-length messages *m*_0_ and *m*_1_. B chooses a random bit *b*∈(0,1} and encrypts *m*_*b*_ under $A^{*}$, then send the challenge ciphertext *CT*′ to A.
**Phase2**: The same as **Phase1**, with the restriction that S does not satisfy $A^{*}$.
**Guess**: A outputs a guess bit *b*′∈{0,1}. If *b*′ = *b*, he wins the game, otherwise, he fails.
Therefore, the $A^{\prime }s$ advantage is defined to be $Adv_{A}=\left|\mathrm{Pr}\left[b^{\prime }=b\right]-\frac{1}{2}\right|$.

## 4. System framework

**Fig 3:**
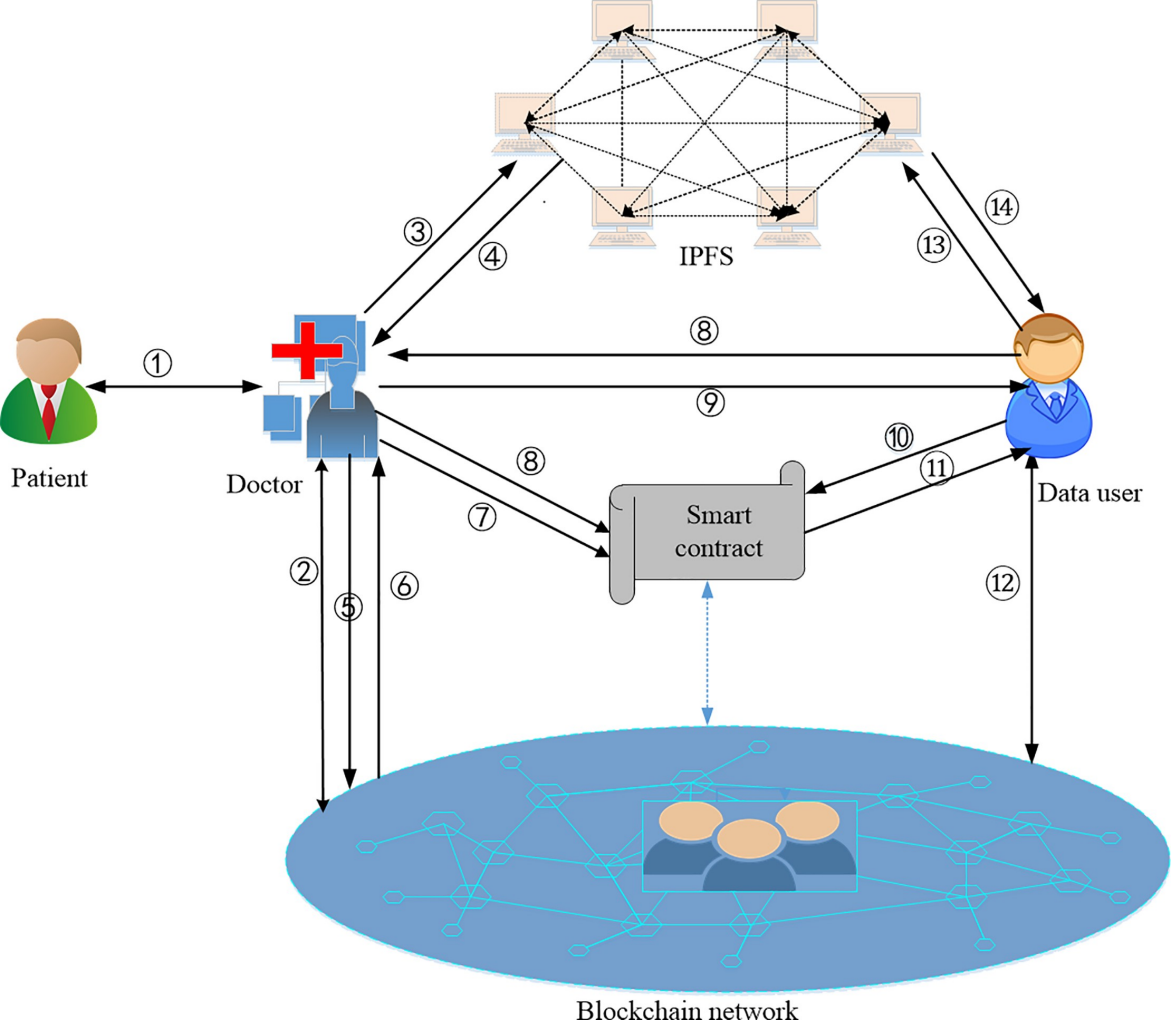
System model.

In this section, we briefly describe the system framework of our scheme. As shown in Fig 3, the system mainly includes five entities: patient, doctor, data requester, IPFS, and blockchain network. Possible interactions between different entities in the paper are shown in Fig 3. Below we discuss the roles of each entity in the system and their interactions.

### 4.1 Roles of each entity

#### Patient

Patient is also a data user, suppose a patient i∈U registers with the hospital k∈H to see a doctor j∈D. The server of the hospital *k* generates a certificate *φ*_*i*_ for the patient and returns it to the patient. At the same time, the server stores *μ*_*i*_ = *H*(*φ*_*i*_) on the service registration list of the doctor *j*. Here *φ*_*i*_ is equivalent to a number plate for the doctor to generate medical information for patient *i*. When the patient goes to the doctor, he will give the doctor a certificate *φ*_*i*_ to generate his personal medical information and access his historical medical records.

#### Doctor

The doctor is responsible for generating medical information for the patient and extracting a set of keyword for the patient’s medical information after obtaining the patient’s consent. In the scheme, doctor is assumed to be honest and not to collude with data requester to get patients’ medical records for illegal benefit. Furthermore, in order to safely store interoperable data, the patient and the doctor discuss an access policy to encrypt the medical information and the keyword to generate the ciphertext. The ciphertext is composed of two parts, one is the ciphertext of the patient’s medical information that is stored on the IPFS, and the other part is the ciphertext of the keyword, which is stored on the smart contract.

#### Data requester

The data requester is the data user, such as scientific research institutions, medical insurance companies, patients’ families, etc., which can obtain relevant records as long as their attributes meet the corresponding access policy.

#### IPFS

The IPFS stores the ciphertext of the patient’s medical information encrypted by the doctor and then returns the corresponding hash value to the doctor, where the hash value is equivalent to a file address. Once the address is obtained, the authorized data requester can download the ciphertext according to the address.

#### Blockchain network

The blockchain network is the core of our paper, and it performs smart contracts in a distributed manner without relying on central entities, which is necessary to ensure the secure storage and sharing of electronic medical information. In order to improve system efficiency, we use the Ethereum blockchain. In our scheme, we do not consider the miners on the Ethereum blockchain for the time being.

### 4.2 The interaction process in Fig 3

Fig 3 is a major system model of our scheme. Below we briefly analyze each step in the model.

① The patient goes to the hospital to see a doctor and interacts with the doctor to generate his medical data.
② The doctor deploys a smart contract on the blockchain, and then generates a hash value of the signed medical data and a message authentication value stored on the blockchain.
③ The doctor encrypts the medical record under the access policy negotiated with the patient and uploads it to IPFS.
④ IPFS returns the location to the doctor where the medical records are stored.
⑤ The doctor encrypts the medical record location, then embeds the ciphertext into the transaction and broadcasts the transaction to the blockchain.
⑥ When the transaction is confirmed, the doctor records the transaction address.
⑦ The doctor encrypts the keywords of the medical records, generates a keyword index, and then stores the transaction address and keyword index into the smart contract.
⑧ The data requester sends a medical record access request to the doctor, and the doctor authenticates the identity of the data requester and adds it to the authorized user of the smart contract.
⑨ The doctor distributes the appropriate attributes as well as generates his attribute key, which is then returned to the data requester through a secure channel.
⑩ The data requester creates a search token and then invokes the smart contract with the token as a parameter.
⑪ The smart contract judges the identity of the data requester. If he is an authorized user, then smart contract returns the relevant search results to him.
⑫ The data requester reads the transaction information from the blockchain and calculates the file location.
⑬ The data requester obtains the file location and then downloads the encrypted medical record from the IPFS.
⑭ The data requester judges whether his attributes satisfy the access structure in the ciphertext, and if so, decrypts and obtains the medical record.

### 4.3 Protocol description

**Fig 4:**
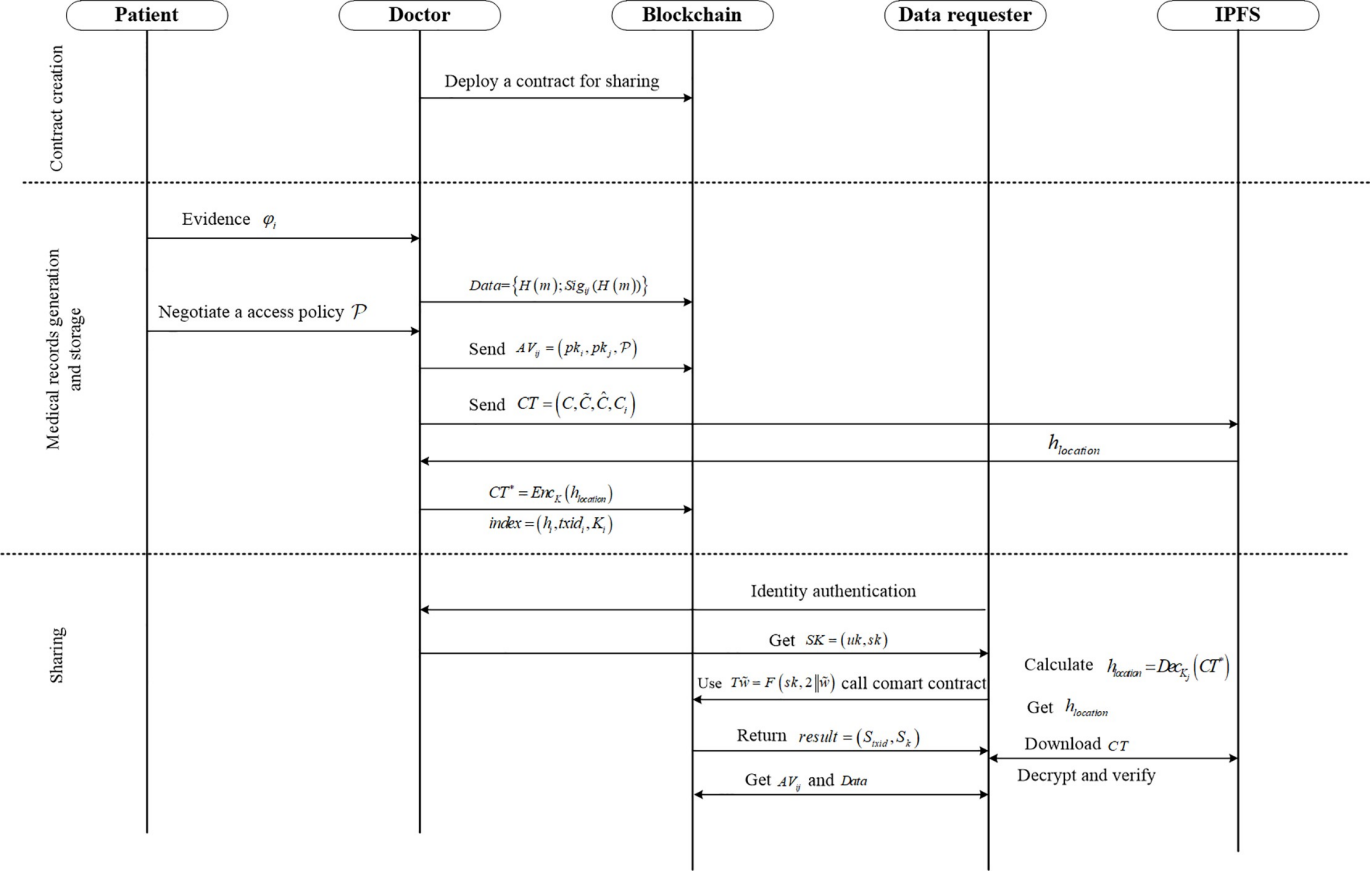
Protocol flow chart.

A sketch of the possible interactions between the different system entities is shown in Fig 4. Our proposed framework consists of three parts: system establishment, medical data generation and storage, data search and access. Each part in the system is discussed in the following paragraphs.

#### Step 1: System establishment

In the first place, the doctor generates the system public key *PK* by implementing the function **GlobalSetup(λ)**, where *λ* is the safety parameter. And each patient and doctor generates their own public and private keys by manipulating function **KeyGen(PK)**. It including three sub-algorithms:

- **GlobalSetup(λ)**: Let *U* be the attribute universe, *g*_0_ be the generator of $G_{0}$, and bilinear map $e:G_{0}\times G_{0}\to G_{1}$. The doctor selects two random numbers $\alpha ,\beta \in {\mathbb{Z}}_{p}^{*}$ and a hash function $H:{\left\{0,1\right\}}^{*}\to G_{0}$, calculates *A* = *e*(*g*_0_,*g*_0_)^*α*^, $B=g_{0}^{\beta }$, and then defines a pseudo-random function *F*:{0,1}^*λ*^×{0,1}*→{0,1}^*λ*^. The public key is published as $PK=\left(G_{0},G_{1},g_{0},e,H,A,B,F\right)$, the master key is *MSK* = (*α*,*β*), and then randomly selects $\xi \in {\mathbb{Z}}_{p}^{*}$ and sets the search private key *sk*_*u*_ = {*ξ*}. Hereafter, the doctor deploys a smart contract on the Ethereum blockchain, which is used to store the encrypted keyword index and provide search services for the data requester. After the contract is successfully deployed, the doctor records the contract’s account address and the contract’s ABI.
- **KeyGen**_**i**_ (**PK**): Each patient i∈U randomly selects the number $y_{i}\in {\mathbb{Z}}_{p}^{*}$ as his private key *sk*_*i*_, and then outputs the public key $pk_{i}=g_{0}^{y_{i}}$.
- **KeyGen**_**j**_ (**PK**): Each doctor j∈D randomly selects the number $x_{j}\in {\mathbb{Z}}_{p}^{*}$ as his private key *sk*_*j*_, and then outputs the public key $pk_{j}=g_{0}^{x_{j}}$.

#### Step 2: Medical data generation and storage

When a patient *i* registers with the hospital *k*, the hospital's server randomly selects $\varphi_{i}\in {\mathbb{Z}}_{p}^{*}$ and sends *φ*_*i*_ securely to patient *i*. At the same time, the server selects a doctor *j* for the patient (the doctor can also choose the patient himself, which is equivalent to the registration in our current medical system), then the server calculates *μ*_*i*_ = *H*(*φ*_*i*_). When the patient goes to see the doctor, he presents *φ*_*i*_ as his evidence and the doctor generates his medical records *m*. After patient *i* interacts with doctor *j*, the doctor would do several steps as follow: (1) extracts the keywords from the medical records to form a set of keywords *W*_*m*_, and then the doctor negotiates an access policy with the patient for the medical record *m*; (2) generates data *Data* = {*H*(*m*);*Sig*_*ij*_(*H*(*m*))} that needs to be saved on the blockchain, where symbol *H*(*m*) represents hash the medical record *m*, symbol *sig*_*ij*_(*H*(*m*)) represents the hash value of medical records signed by the doctor and the patient using their private keys *sk*_*i*_ and *sk*_*j*_ respectively; (3) generates a message authentication value $AV_{ij}=\left(pk_{i},pk_{j},P\right)$, where *pk*_*i*_ and *pk*_*j*_ represent the public key of the patient and the doctor, respectively, and P represents a negotiated access policy; (4) creates a data store transaction, then broadcast data *Data* = {*H*(*m*);*Sig*_*ij*_(*H*(*m*))} and authentication value $AV_{ij}=\left(pk_{i},pk_{j},P\right)$ to the blockchain network.

Note: This signature is signed by the doctor and patient commonly to ensure the accuracy and authenticity of the medical record. That is to say, to prevent the doctor tampers with the medical record before encrypting it, a data requester can obtain *Data* and *AV*_*ij*_ from the blockchain network to verify the integrity of the medical record when he obtains the medical record.

Finally, the doctor encrypts the patient’s original records *m* and keyword sets *W*_*m*_ using the following two sub-algorithms based on the access policy negotiated with the patient.

- **Record-Encrypt(K)**: The encryption algorithm selects a random number $s\in {\mathbb{Z}}_{p}^{*}$ and sets *C* = *m*⋅*A*^*s*^, $\tilde{C}=B^{s}$, $\hat{C}=g_{0}^{s}$. The access policy negotiated between the doctor and the patient is AND gate $A=\left(A_{1},A_{2},\cdots ,A_{n}\right)$, for each attribute in the AND gate A, sets $C_{i}=H{\left(A_{i}\right)}^{s}$, (*i* = 1,⋯,*n*). The ciphertext is $CT=\left(C,\tilde{C},\hat{C},C_{i}\right)$, then the doctor uploads the *CT* to IPFS, and records the file location *h*_*location*_ returned by IPFS. In addition, to encrypt the file location *h*_*location*_, the doctor randomly selects the AES key *K*, calculates CT* = *Enc*_*K*_(*h*_*location*_), then embeds CT* into the transaction *TX*_*ct*_, and broadcasts *TX*_*ct*_ to the blockchain. When the transaction *TX*_*ct*_ is confirmed, the transaction *txid* id and the corresponding encryption key *K* are recorded.
- **Index-Gen**:The keyword index generation algorithm is run by the doctor. For each keyword *w*_*i*_∈*W*_*m*_ extracted from the medical record *m*, the doctor calculates *h*_*i*_ = *F*(*sk*_*u*_,1‖*w*_*i*_), *n*_*i*_ = *F*(*sk*_*u*_,2‖*w*_*i*_), *txid*_*i*_ = *n*_*i*_⊕*txid*, *K*_*i*_ = *n*_*i*_⊕*K*, and then stores the keyword index *index* = (*h*_*i*_,*txid*_*i*_,*K*_*i*_) to the smart contract.

#### Step 3: Medical data search and access

If the patient goes to see the doctor again, he can show *φ*_*i*_ directly, and the doctor will treat him by calculating *μ*_*i*_ = *H*(*φ*_*i*_). If the patient is going to see a doctor across a hospital, or a research institution wants to obtain the medical data for research, or other legitimate data requester wants to access the medical data, they can obtain the medical data through the following four sub-algorithms:

- **Key-Generation**: When a data requester needs to obtain medical record of the patient, he first sends an access request as well as submits his Ethereum account public key to the doctor. The doctor authenticates the identity of the data requester and distributes the appropriate attributes $S=\left(att_{1},att_{2},\cdots att_{n}\right)$ to him, then adds his account address to the authorized user set of the smart contract. Besides, the key generation algorithm generates attribute secret key and search secret key. The algorithm randomly selects elements $r_{i}\in {\mathbb{Z}}_{p}\left(i=1,2\cdots ,n\right)$, sets $r=\sum_{i=1}^{n}r_{i}$, $\tilde{D}=g_{0}^{\frac{\alpha +r}{\beta }}$. For each attribute $att_{i}\in S$, randomly choose $r^{\prime }\in {\mathbb{Z}}_{p}$, let $D_{i}=g_{0}^{r_{i}}\cdot H{\left(att_{i}\right)}^{r^{\prime }}$, $\hat{D}=g_{0}^{r^{\prime }}$. The attribute secret key and search secret of data requester are defined as $uk=\left(\tilde{D},\hat{D},\left\{D_{i}|i=1,2,\cdots ,n\right\}\right)$ and {*sk* = *ξ*} respectively, where the search secret key is same for different data requester. The doctor then sends the secret key *SK* = (*uk*,*sk*) and the address of the smart contract to the data requester via a secure channel.
- $TokenGen\left(\tilde{w},sk\right)$: The algorithm is run by the data requester, he creates a search token for his chosen keyword $\tilde{w}$ from the medical record and his search secret key *sk* as: $T\tilde{w}=F\left(sk,2\|\tilde{w}\right)$. Hereafter, he uses the token as a parameter to invoke the smart contract.
- **Test**: The test algorithm is run by the smart contract. Firstly, the smart contract judges whether the data requester is an authorized user. If not, the search operation is refused, otherwise the search result *result* = (*S*_*k*_,*S*_*txid*_) is returned according to the token, where *S*_*k*_ is the corresponding encrypted *K* set and *S*_*txid*_ is the encrypted *txid* set.
- **Decrypt**: The data requester obtained the search result from the smart contract, he calculates $n=F\left(sk,2\|\tilde{w}\right)$. For $txi{\tilde{d}}_{j}\in S_{txid}$, *K*_*j*_∈*S*_*k*_, he calculates $txid_{j}=n⊕txi{\tilde{d}}_{j}$, $K_{j}=n⊕{\tilde{K}}_{j}$. Then data requester reads the transaction data *txid*_*j*_ from the Ethereum blockchain and calculates $h_{location}=Dec_{K_{j}}\left(CT^{*}\right)$, where $Dec_{K_{j}}\left(CT^{*}\right)$ indicates that the AES algorithm is used to decrypt CT* and the decryption key is *K*_*j*_. The data requester decrypts and obtains *h*_*location*_, downloads the encrypted medical record from the IPFS, and then to judge whether the attribute satisfies the access policy, that is $att_{i}=A_{i}\left(i=1,2,\cdots n\right)$. If not satisfied, he returns to terminate and reads the next transaction data. Otherwise, the medical record *m* is restored as $E=\prod_{i=1}^{n}\frac{e\left(D_{i},\hat{C}\right)}{e\left(\hat{D},C_{i}\right)}=e{\left(g_{0},g_{0}\right)}^{rs}$, the algorithm is decrypted by calculation $m=C/\left(e\left(\tilde{D},\tilde{C}\right)/E\right)$.

### 4.4 Application of our scheme: Insurance claims

**Fig 5:**
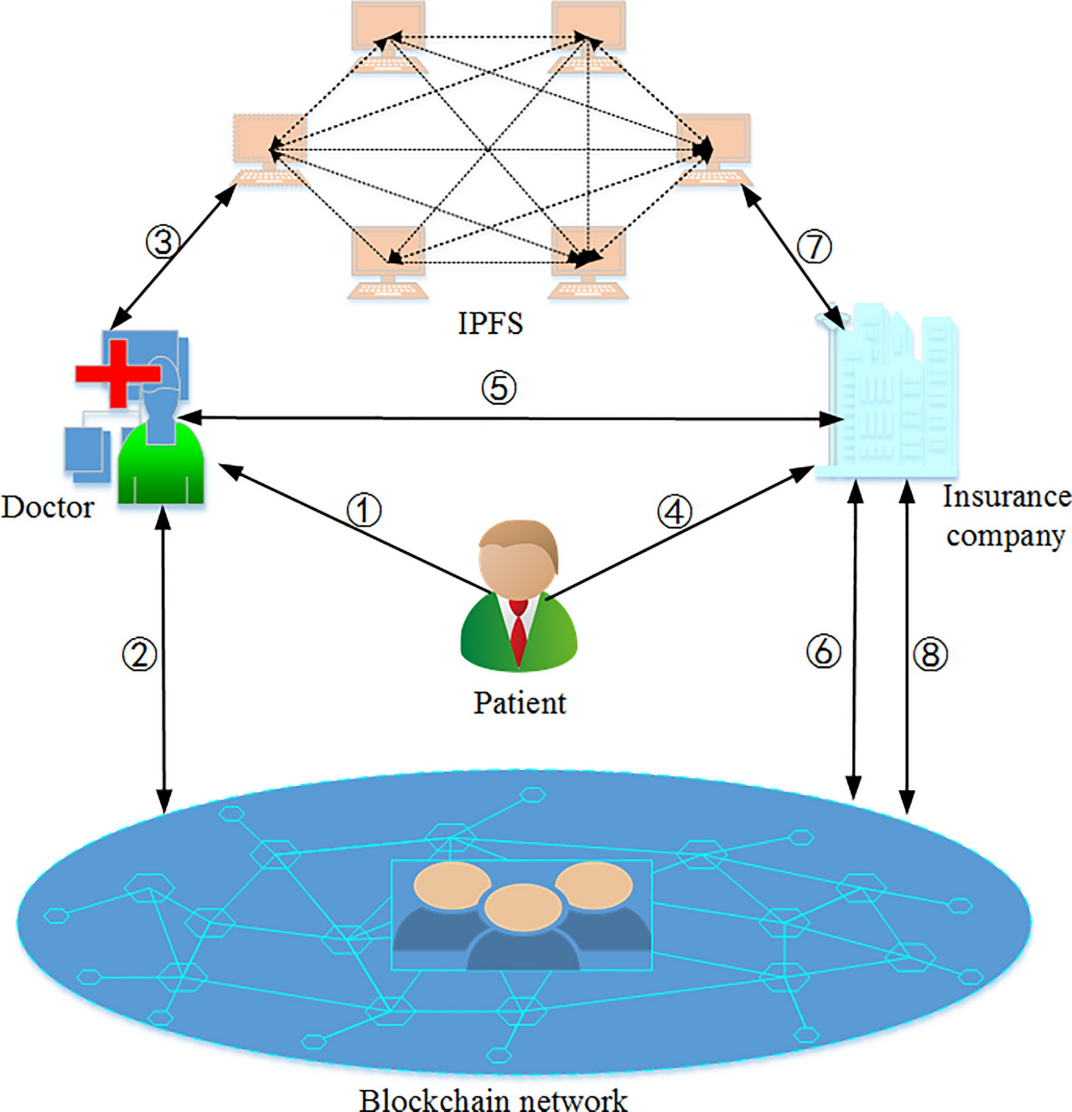
Insurance claims.

In recent years, with the development of medical insurance business, the number of participants has increased, and insurance claims have also increased. However, in the real insurance claims process, there are lots of medical insurance cases that cheat the insurance companies to make compensation by falsifying medical records. The medical record is a document that truly records the patient’s health information; it is also an significant evidence for the insurance company to pay for medical costs when conducting audit claims. In fact, there are some problems in traditional medical record management methods and some understandings in the authenticity of medical records. For example, after the patient and some members of the hospital reached an agreement, the doctor or the hospital alone forged the patient’s medical information and then illegally received the claim. Based on the above issues, the completeness, timeliness, and especially authenticity of medical records must be ensured. What's more, a proper solution is provided from our framework. Take medical insurance as an example, the process involves three entities: insurance company, patient and hospital. The specific workflow is shown in Fig 5.

① The patient negotiates with the doctor about the medical record access structure, including allowing the insurance company to access the data.
②③ The doctor encrypts the medical record based on the negotiated access structure, writes the relevant description of the medical record into the blockchain, and stores the encrypted complete initial medical record on the IPFS.
④⑤⑥⑦ The patient filed a claim with the insurance company. After receiving the application, the insurance company will conduct identity authentication to the doctor to obtain the relevant private key, and then generate the search token with the corresponding keywords to invoke the smart contract, so as to obtain the address information of the medical record in the IPFS from the blockchain. Finally, the corresponding encrypted medical record can be downloaded and decrypted using its secret key.
⑧ Insurance company interacts with the blockchain to verify the integrity and authenticity of the decrypted data. If the medical record is true and accurate, the insurance company completes the claim.

### 4.5 Smart contract creation

The doctor deploys a smart contract by broadcasting a transaction to the blockchain network. The deployed contract is described in the algorithm 1 and contains the following principal features:

#### Add authorized users

When a data requester needs to obtain medical record of the patient, he first sends an access request as well as submits his Ethereum account public key to the doctor. The doctor certificates the identity of the data requester and then adds his account to the smart contract of the authorized.

#### Search medical records

The keyword index of the medical record is stored on the smart contract. And the data requester generates a search token to invoke the smart contract, then the contract first judges whether the data requester is an authorized user, and if so, returns the relevant seek result; otherwise, the operation is terminated. This operation can avoid the drawbacks of data storage on the cloud server. In other words, the cloud server is semi-honest and curious, he will return incorrect or partial search results to the data requester during the search process.

**Algorithm 1:Pseudocode for the EMRs Sharing contract**

  **contract** EMRs Sharing //Doctor

    address public Doctor;

    **mapping**(address = >bool) public authorizeRequesters;

    // requester authorize

    **mapping**(bytes = >indexSet[]) Index;

    // storage index

    **event** Searchevent (bytes key, bytes result);

    **function** addRequester (address newRequester) onlyDoctor() public

        **if** (!authorizeRequesters[newRequester])

          authorizeRequesters[newRequester] = true;

        **end**

        **return** authorizeRequesters[newRequester];

**function** addIndex (keywordIndex, txid, key) public

        **if** emr.sender is not doctor **then**

          **throw**

        **end**

          **mapping** keywordIndex to (txid, key), and add it to index variable collection;

        **return** True

**function** search(Token) haveAuthorized(Requesters) public

        **if** *tx*.*origin is not Doctor* **then.**

          **throw**

        **end**

        **if** Requester not in authorizeRequesters list **then**

          **throw.**

        **end**

        length = Index[Token].length;

        result = new strings.slice[](length);

        result1 = new strings.slice[](length);

        **for** i = 0; i < length; i++ **do**

          result[i] ← (Index[keywordHash][i].key);

          result1[i] ← (Index[keywordHash][i].txid);

        **end**

          Searchevent(emr.sender, result, result1);

          **return** (result, result1)

## 5. Security

### 5.1 Security analysis

This part conducts a security analysis on the proposed scheme from three aspects: secure storage, privacy protection and tamper-proof.

#### Secure storage

The storage security of data is an important feature of this paper. The whole process of production and use of the EMRs is secure in the scheme. Public information on medical records is stored on the blockchain and cannot be tampered with and publicly visible. As the medical record producer, the doctor performs a hash operation on the medical record and stores it on the blockchain. The original medical records are then encrypted and stored in the IPFS and the location returned by the IPFS is encrypted and written into the blockchain, ensuring the authenticity and confidentiality of the data source. Furthermore, the distributed storage characteristics of IPFS guarantee the security of the medical records storage.

#### Privacy protection

In the first place, the data requester participate in the transaction on the blockchain with an anonymous way, and each transaction can output different public-private key pairs, which effectively protects the identity information of the data requester to a certain extent. Secondly, the public hyperledger does not contain the patient’s privacy information, and the complete medical record is encrypted and stored in the IPFS. The producer of the medical data maybe retain the raw data of the patient’s medical record, which requires related mechanisms and laws to prevent doctors and medical institutions from revealing the user’s medical record to protect the patient’s privacy. Thirdly, only the address information of the ciphertext is stored in the blockchain, and the unauthorized data requester cannot obtain the corresponding location. In addition, if the attributes of data requester don’t satisfy the access policy embedded in the ciphertext, and the plaintext information of the medical record cannot be obtained, so it is impossible to get any actual data about the medical record from the public information of the blockchain.

#### Tamper-proofing

All information on the blockchain is public, tamper-proof and in a chronological order. The distributed consensus mechanism on the blockchain makes its trust based on cryptographic algorithms without relying on trusted third provider. Once the data is written into the blockchain, it cannot be tampered with, because each block is saving the hash value of its previous block. If one want to modify the data of a block, needing at least 51% of the total calculating power, which is almost impossible. The hash value of the original data of the medical record is preserved in the blockchain, and any change of the original data will cause a change of its hash value, so it also directly guarantees the inelastic non-tampering of the medical record.

### 5.2 Security proof

**Theorem 1.** *In the general group model, for any adversary A, let q be a bound on the sum number of group elements it receives from queries it makes to the oracles for the hash function, groups $G_{0}$ and $G_{1}$, and the map e, and from its interaction with the IND-CPA security game. Then we have that the advantage of the adversary in the IND-CPA security game is $O\left(q^{2}/p\right)$.*

**Proof.** The initialize *g* = *ϕ*_0_(1), *g*^*x*^ = *ϕ*_0_(*x*), that is to write *e*(*g*,*g*) = *ϕ*_1_(1), *e*(*g*,*g*)^*x*^ = *ϕ*_1_(*x*). The adversary A will interact with the challenger B using the *ϕ*- denotes of the group elements. In the security game B communicates with A as follows:

**Setup**: Challenger B randomly selects $\alpha ,\beta \in {\mathbb{Z}}_{p}^{*}$, and calculates the public key is $PK=\left(A=e{\left(g_{0},g_{0}\right)}^{\alpha },B=g_{0}^{\beta }\right)$, the master key is *MSK* = (*α*,*β*), then sends the public key *PK* to the adversary A, and keeps the master key *MSK* privately.

When the adversary (or challenger) invokes for the evaluation of *H* on any attribute *att*_*i*_, a new random number *t*_*i*_ is selected from ${\mathbb{Z}}_{p}^{*}$, and the simulation provides $g_{0}^{t_{i}}$ as the response to *H*(*att*_*i*_).

**Phase1**: A sends a query to the $O_{SK}\left(S\right)$ oracle:

$O_{SK}\left(S\right)$: The adversary A issues attribute private key query to the set $S=\left(att_{1},att_{2},\cdots ,att_{n}\right)$. The challenger B selects a new random value $r\in {\mathbb{Z}}_{p}^{*}$, calculates $\tilde{D}=g_{0}^{\frac{\alpha +r}{\beta }}$. And for each attribute in $att_{i}\in S_{i},i=\left(1,\cdots ,n\right)$, selects new random numbers *r*_*i*_, *r*′ from ${\mathbb{Z}}_{p}^{*}$, sets $D_{i}=g_{0}^{r_{i}+t_{i}r^{\prime }}$, $\hat{D}=g_{0}^{r^{\prime }}$. The attribute private key is defined as $uk_{att}=\left\{\tilde{D},D_{i},\hat{D}\right\}$. Then sends the private key to adversary A.

**Challenge**: A generates a pair of messages *m*_0_ and *m*_1_, hoping to challenge it. In addition, A announced a challenge access structure A. The challenger B creates a challenge ciphertext, he first selects a random number $s\in {\mathbb{Z}}_{p}^{*}$ and sets *C* = *m*_*b*_⋅*e*(*g*_0_,*g*_0_)^*αs*^, $\tilde{C}=g_{0}{}^{\beta s}$, $\hat{C}=g_{0}^{s}$. Hereafter, for each attribute in the AND gate, B sets $C_{i}=g_{0}^{t_{i}s}$, *i* = (1,2,⋯,*n*), and then these values are sent to A.

**Phase2**: The same as **Phase1**, with the restriction that S does not satisfy $A^{*}$.

**Guess**: A returns a guess *b*′∈{0,1}.

We can consider an improved game where the truly challenging ciphertext is replaced by *e*(*g*,*g*)^*θ*^ instead of *m*_*b*_⋅*e*(*g*,*g*)^*αs*^, and the probability of distinguishing *m*_*b*_⋅*e*(*g*,*g*)^*αs*^ from *e*(*g*,*g*)^*θ*^ is equal to half the probability of distinguishing *m*_1_⋅*e*(*g*,*g*)^*αs*^ from *m*_0_⋅*e*(*g*,*g*)^*αs*^.

Next, a detailed shows of the $B^{\prime }$ simulation is depicted. If there is no "accidental collision" that the $B^{\prime }$ simulation is perfect. More specifically, we think of an oracle query as being a rational function *δ* = *η*/*ξ* in the variables *θ*,*α*,*β*,*r*,*r′*,*r*_*i*_,*s*. An unexpected collision would be when two queries corresponding to two distinct formal rational functions *δ* = *η*/*ξ*, *δ*′ = *η*′/*ξ*′, but where since the random selects of these variables’ values, we have that the values of *δ* = *η*/*ξ* = *η*′/*ξ*′ = *δ*′, where *η*≠*η′*, *ξ*≠*ξ*′.

This unexpected collision does not occur in group $G_{0}$ or $G_{1}$ for our now condition. For any pair of queries corresponding to different rational functions *η*/*ξ* and *η′*/*ξ′*, a unexpected collision only occurs when there is a non-zero multiple *ηξ*′−*ξη*′ = 0. According to the literatures [34], this event’s probability is O(1/p). And then in the constraint conditions, the probability of accidental collision happens is at most $O\left(q^{2}/p\right)$.

Now we state that even if B replaces *αs* with the variable *θ*, the A‘s view would have been identically distributed. Due to *θ* only appears as *e*(*g*,*g*)^*θ*^, if a collision occurs, we have *δ*−*δ*′ = *γαs*−*γθ*, where *γ*≠0 is a constant. Then we can artificially add query *δ*−*δ*′+*γθ* = *γαs* to the A‘s query. But now we need to prove that the A can never build a query for *e*(*g*,*g*)^*γαs*^. To build the item *αs*, the A can pair *sβ* with (*a*+*r*)/*β*. In this way, the adversary would found a query polynomial containing *γαs*+∑*γ*′*sr*. In order to get an inquiry from *γαs*, the adversary A must eliminate the item of the form ∑*γ*′*sr*. However, according to the simulation, the adversary A cannot get the master key. Therefore, the adversary A cannot get the form *αs* and construct a query for *e*(*g*,*g*)^*γαs*^.

## 6. Performance evaluation

In this section, we provide performance evaluation of the proposed scheme. Firstly, we compare the security properties among the proposed protocol and several other literatures. Later, the computational overhead of the cryptographic operations are analyzed. Finally, we evaluated the performance in the cost of smart contract and the system data throughput.

### 6.1 Comparison of schemes characteristics

We have chosen the recently proposed medical record sharing schemes [5, 8, 10, 12, 25], as a benchmark. Table 2 compares some of the features of the blockchain-based protocols Peterson [8], Xia [10], and non-blockchain based protocols Au [5], Yang [12], and Sun [25]. As it can be seen from the table, Yang [12], Sun [25] and our proposed scheme achieve searchability. It is worth noting that these schemes have the characteristics of privacy protection and access control, which is a key security goal of the electronic record sharing system. In addition, regardless whether it is based on the blockchain, only in our scheme, the data is stored in the IPFS, which effectively solves the problem that the data lost or tampered in the cloud environment. And it is fortunate that only our scheme meets all the properties, and our scheme more suitable for current computing systems and practical applications.

**Table 2:** Comparison of schemes properties.

Scheme	Blockchain-Based	Access control	Secure search	Privacy protection	Storage environment
*[5]*	×	√	×	√	Cloud
*[8]*	√	√	×	√	—
*[10]*	√	√	×	√	Cloud
*[12]*	×	√	√	√	Cloud
*[25]*	×	√	√	√	Cloud
*Ours*	√	√	√	√	IPFS

### 6.2 Computational complexity analysis

We present some major time-consuming operational symbols, exponential operations *E*, and bilinear pairwise operations *P*, before analyzing the computational complexity. Since our scheme is based on ABE construction, we use *n*_*a*_ to represent all the number of attributes in the scheme, and *n*_*a*,*u*_ to represent the number of attributes owned by a data requester in the scheme. As it can be seen from Table 3, in the global setup phase, our computational overhead requires only one pair of operations and three exponential operations, and is a constant. In the EMRs encryption phase, since the corresponding access policy is set, the required computing operations are related to the number of attributes. During the user key generation phase, the doctor distributes the attribute private key to the data requester, and the required operations are also related to the number of attributes. In the decryption phase, the data requester needs to calculate two pairs of operations related to the number of attributes.

**Table 3:** Calculation cost.

Algorithm	Setup	Record-Encrypt	Key-Generation	Decrypt
Calculation cost	3*E*+*P*	(*n*_*a*_+2)*E*+*P*	(*n*_*a*,*u*_+2)*E*	2*n*_*a*,*u*_*P*

### 6.3 Time cost of cryptographic algorithm

**Fig 6:**
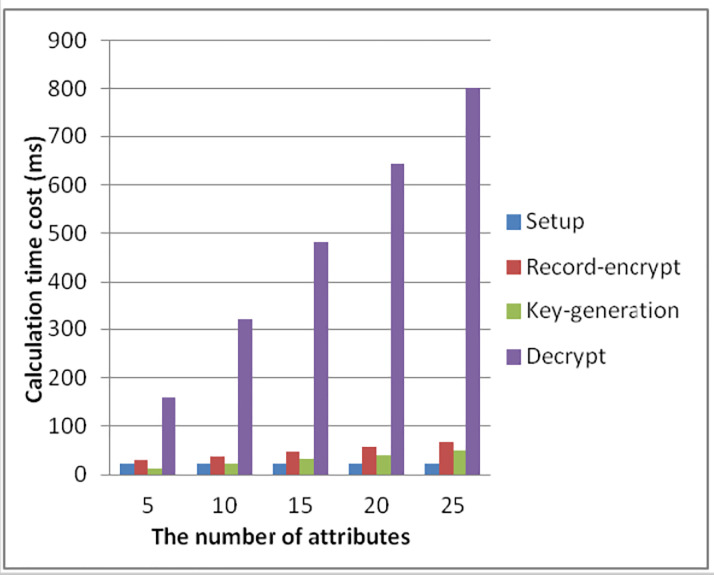
Calculation time cost under different number of attributes.

In order to analyze the actual time overhead of the cryptosystem, a series of simulation experiments were carried out using a actual data set and a PBC (Pairing-Based Cryptography) library [35]. Because the time cost of some cryptographic algorithms varies with the number of attributes, we simulate some time costs for different number of attributes. In order to better meet the current actual needs, we assume that the number of attributes is |*n*_*a*_| = |*n*_*a*,*u*_|∈[5,25], and the experimental results are shown in Table 4 and Fig 6.

**Table 4:** Time cost (in ms) of cryptographic algorithms.

The number of attributes	Algorithm	Calculate time cost (ms)
	Setup	21.71
	Record-Encrypt	29.238
5	User-Request	13.174
	Decrypt	160.64
	Setup	21.71
	Record-Encrypt	38.648
10	User-Request	22.584
	Decrypt	321.28
	Setup	21.71
	Record-Encrypt	48.058
15	User-Request	31.994
	Decrypt	481.92
	Setup	21.71
	Record-Encrypt	57.468
20	User-Request	41.404
	Decrypt	642.56
	Setup	21.71
	Record-Encrypt	66.878
25	User-Request	50.814
	Decrypt	803.2

We separately analyzed the time cost of some encryption algorithms in the article when the number of attributes is 5, 10, 15, 20, 25. As it can be seen from Table 4 and Fig 6, in the establishment phase of the cryptographic algorithm, with the number of attributes changing, the required time cost is always keep a constant of 21.71 ms, since there is no attribute operation involved in this stage. In the medical record encryption algorithm, when the number of attributes is 5, 10, 15, 20, 25, the time required by the algorithm is 29.238 ms, 38.648 ms, 48.058 ms, 57.468 ms, 66.878 ms, respectively. Compared with the medical record encryption algorithm, the user request algorithm reduces the required time by about 16.064 ms under the corresponding number of attributes. As it can be seen from Fig 6, in the decryption algorithm, since our scheme involves two pairs of operations, the time cost required is slightly longer than other algorithms.

### 6.4 The cost of smart contract

In order to test the gas cost of the smart contract, we implement it on intel(R) Core(TM) i5-2450M CPU2.50GHz and 4.00GB of RAM with Ethereum in solidity [36] code, a programming language for writing contracts on Ethereum. We tested the cost of deploy contract and add requester. In the experiment, we set *gasprice* = 1*Gwei*. The cost of gas used in smart contract is shown in Table 5. As can be seen from Table 5, the deploy contract operation is performed during the system establishment phase at a cost of 0.000154455 Ether. The add requester operation is performed during the medical data search and access phase, and the operation is performed after the doctor authenticates the identity of the access requester, and the cost of the operation is 0.000047869 Ether.

**Table 5:** Smart contract cost test (gas price = 2 Gwei).

Function	Gas used	Gas cost (ether)
Deploy contract	772275	0.000154455
Add requester	23948	0.000047869

By analyzing the test results in Table 5, it is not difficult to find that our electronic medical record sharing contract requires less cost in deployment and invocation, and it is acceptable for users. In addition, although the cost of calling some of the functions in the contract increases as the number of electronic medical records increases, the increase is small. Therefore, our scheme is feasible in practice.

### 6.6 System data throughput analysis

In the scheme, the block is composed of a block header and a block body. The size of the block header is about 80 bytes, and a certain number of transactions make up a block body. By analyzing the data, it is concluded that the size of a transaction is 256 bytes. We assume that a block contains 20 transaction information, the size of a block is calculated as 256×20+80 = 5200*bytes*≈5*Kb*, on this basis we can measure the throughput of the network.

Suppose an EMRs system with 500 users, where the peak number of transactions generated per second is 50 users sending transaction information. Therefore, the throughput of system data can be calculated as follows:

50×(5200)=260000bytes/s≈253.9Kb/s

50×(5200×60)=15600000bytes/min≈14.98Mb/min

50×(5200×60×60)=936000000bytes/h≈892.64Mb/h

50×(5200×60×60×24)=22464000000bytes/day≈20.92Gb/day

According to the data calculated above, we can calculate the total amount of data of the system over a period of time, so as to estimate the growth of block chain data. The results are shown in Table 6, where the ordinate and abscissa respectively represent the number of transactions and a fixed period of time. The data in the table is calculated based on the number of peak transactions in the network.

**Table 6:** Total amount of system data in a fixed time.

Tansaction amount	1 s	1 h	1 day	1 year
500	2.479Mb	8.717Gb	209.21Gb	74.57Tb
5000	24.79Mb	87.17Gb	2.04Tb	744.6Tb
50000	247.94Mb	871.72Gb	20.43Tb	7.28Pb
500000	2.42Gb	8.51Tb	204.3Tb	72.8Pb

Based on the data calculated above, we can deduce the total amount of data of the system over a period of time, so as to estimate the growth of the amount of data on the blockchain. The results are shown in Table 6, where the ordinate and abscissa represent the number of transactions and a fixed period of time, respectively. The data in the table is calculated based on the number of peak transactions in the network. Compared with the Bitcoin system, in the Bitcoin system, when the transaction volume is 2000, the amount of data generated in one year can reach the Pb level. However, in our system, when the transaction volume reaches 10,000, the amount of data generated in a year can reach the Pb level. Therefore, from the perspective of the amount of data, our scheme has more advantages.

## 7. Conclusion

We have proposed a framework using blockchain and smart contract technology to solve the problem of secure storage and sharing of current EMRs. The system enables doctors firstly encrypt electronic medical records with appropriate access policies and then upload the ciphertext to IPFS. The combination of IPFS and blockchain allows doctors to process large amounts of electronic medical data via IPFS, to eliminate the need of putting the data itself on the chain, to save network bandwidth in the blockchain. While using doctors and patient encryption to achieve electronic medical record security, a keyword index for searching encrypted medical records is also designed. The encrypted keyword index data is stored in the Ethereum blockchain, and the smart contract is deployed on the Ethereum blockchain to perform keyword search in the distributed system. And we use ABE to achieve fine-grained access control for data requester. Security analysis shows that the protocol implements data security, privacy protection, and secure search. In addition, we evaluated performance from three aspects: the characteristics of the scheme, the time cost of cryptographic algorithm, and the smart contract cost. In future work, we will address the more complex needs of EMRs system, for instance, multi-keyword search and time-controlled revocation.

## Supporting information

S1 Appendix (DOC)
